# Extracting Coastal Raft Aquaculture Data from Landsat 8 OLI Imagery

**DOI:** 10.3390/s19051221

**Published:** 2019-03-11

**Authors:** Jun Wang, Lichun Sui, Xiaomei Yang, Zhihua Wang, Yueming Liu, Junmei Kang, Chen Lu, Fengshuo Yang, Bin Liu

**Affiliations:** 1 Geological Engineering and Institute of Surveying and Mapping, Chang’an University, Xi’an 710054, China; 2017026007@chd.edu.cn (J.W.); sui1011@chd.edu.cn (L.S.); 2017026008@chd.edu.cn (J.K.); 2 State Key Laboratory of Resources and Environment Information System, Institute of Geographic Sciences and Natural Resources Research, CAS, Beijing 100101, China; yangxm@lreis.ac.cn (X.Y.); liuym@lreis.ac.cn (Y.L.); luchen@lreis.ac.cn (C.L.); yangfs@lreis.ac.cn (F.Y.); liub@lreis.ac.cn (B.L.); 3 Jiangsu Center for Collaborative Innovation in Geographical Information Resource Development and Application, Nanjing 210023, China; 4 University of Chinese Academy of Sciences, Beijing 100049, China

**Keywords:** coastal raft aquaculture, remote sensing, Landsat 8 OLI, NDVI, edge detection, GEOBIA

## Abstract

Information, especially spatial distribution data, related to coastal raft aquaculture is critical to the sustainable development of marine resources and environmental protection. Commercial high spatial resolution satellite imagery can accurately locate raft aquaculture. However, this type of analysis using this expensive imagery requires a large number of images. In contrast, medium resolution satellite imagery, such as Landsat 8 images, are available at no cost, cover large areas with less data volume, and provide acceptable results. Therefore, we used Landsat 8 images to extract the presence of coastal raft aquaculture. Because the high chlorophyll concentration of coastal raft aquaculture areas cause the Normalized Difference Vegetation Index (NDVI) and the edge features to be salient for the water background, we integrated these features into the proposed method. Three sites from north to south in Eastern China were used to validate the method and compare it with our former proposed method using only object-based visually salient NDVI (OBVS-NDVI) features. The new proposed method not only maintains the true positive results of OBVS-NDVI, but also eliminates most false negative results of OBVS-NDVI. Thus, the new proposed method has potential for use in rapid monitoring of coastal raft aquaculture on a large scale.

## 1. Introduction

Aquaculture is one of the fastest growing food production sectors worldwide, an important source of food in many countries, the main protein source for hundreds of millions of people, and has been in the spotlight for its potential to support future food security at a global scale [1]. According to the United Nations Food and Agriculture Organization (FAO) human consumption of farmed aquaculture species exceeded that of capture fisheries for the first time in 2014; in particular, Asia alone generates 90% of the total global aquaculture volume [2]. Coastal raft aquaculture, as one of the most popular industries in coastal areas, plays an important role in regional aquaculture production [3]. However, the negative environmental and social effects of aquaculture have overshadowed the opportunities for employment, income, and foreign exchange from coastal aquaculture. The environmental impacts include mangrove loss, bycatch during collection of wild seed and broodstock, introduction and transfer of species, spread of parasites and diseases, misuse of chemicals, and release of wastes. The socioeconomic impacts include privatization of public lands and waterways, loss of fisheries livelihoods, food insecurity, and urban migration [4]. Moffitt et al. [5] explored ecosystem approaches to aquaculture including addressing climate change, ecosystem restoration, protected areas, and control of invasive species, and then integrated aquaculture assessments and exchanges into an integrated framework. They pointed out that the main challenge of aquaculture governance is to ensure that effective measures are taken to ensure environmental sustainability without undermining aquaculture initiative and social harmony. The FAO and UNEP [6] has pointed out that the effects of pollution from coastal raft aquaculture are limited but incremental and cumulative, and it often takes place in areas where resource ownership or use rights are ill defined and ambiguous. So, in the future development of coastal raft aquaculture areas, selecting suitable aquaculture sites and rationally arranging the spatial distribution of the raft aquaculture areas will help to protect resources in different regions. Gao et al. [7] analyzed the spatial information dynamic remote sensing of the Zhujiang River Estuary aquaculture development, and comprehensively obtained the regional differences in the distribution of the aquaculture areas, which are related to the economic and spatial development needs of different regions. Xia et al. [8] discussed a method for estimating the pollution load of different aquaculture types, and analyze the spatial distribution characteristics in typical bay, taking the Zhelin Bay in Guangdong Province as an example, which provided a scientific basis for pollution monitoring and production regulation of coastal raft aquaculture. All of these are about an important piece of information: the spatial distribution information of coastal raft aquaculture areas.

The Fisheries and Aquaculture Department of the FAO recently stated that remote sensing can provide a promising assessment tool to help estimate the productivity and yield of fisheries [2]. Earth observation by satellite remote sensing holds the potential to fill the need for routine and reliable data on aquaculture at large scales. The free and open data access to long-term missions include the US sensors of the Advanced Spaceborne Thermal Emission and reflection Radiometer, MODerate resolution Imaging Spectroradiometer, and Landsat fleet; European remote sensing satellites include the European Remote Sensing units 1/2, Envisat, and the European Space Agency’s recently launched Sentinels [9]. These missions foster the use of Earth observation data and products for applications in the aquaculture sector. Satellite-derived data products can significantly contribute to large-scale mapping of aquaculture, and help scientists gain a better understanding and management to improve the quantification of coastal raft aquaculture and related production volumes, and also help to ensure the availability of comparable statistics among countries and regions. However, the application of remote sensing to raft aquaculture has lagged behind that of land vegetation and terrain change. The spectral differences between different targets on land are much larger than those between raft aquaculture in water, such as vegetation and non-vegetation areas. Moreover, the raft aquaculture’s spectrum exhibits very complex changes with suspended sediment and chlorophyll concentrations in the background waters [10,11]. These factors have brought great difficulties in extracting coastal raft aquaculture areas from remote sensing images.

Currently, high resolution satellite imagery has been widely used in detecting raft aquaculture areas. For instance, Wang [12] proposed a method of raft aquaculture extraction based on 2 m high resolution images. The method is based on combining linear features with the inclusion relationship of segmented objects. This method provides a high level of accuracy greater than 90% and does not require the collection of samples. Xie et al. [13] used an object-oriented segmentation method to extract the coastal raft aquaculture area from the high resolution satellite imagery of Satellite Pour l’Observation de la Terre or SPOT-5, which is better used in the context of semantic relationships in remote sensing image [14,15,16,17,18,19]. Other researchers [20,21,22,23] have used similar methods. However, the acquisition of high spatial resolution optical satellite imagery is expensive, placing a major constraint when increasing the extraction of aquaculture data on large spatial and time scales. The use of mid-resolution images in the extraction of raft aquaculture areas has also been developed. Li et al. [21] and Wang et al. [24] extracted the lake enclosure culture area using the mid-resolution images, but the results of their output were the border of the aquaculture area. In fact, there are many water areas among the aquaculture areas that are not farmed. To solve this problem, Wu et al. [11] developed a new method based on Landsat 8 data. However, it is a pixel-based method, and the phenomenon of salt and pepper noise is inevitable. And in the target enhancement process, the sample selection is needed, which will lead to greater uncertainty in the result of the algorithm. Xu et al. [25] used the object-based classification method to overcome the problem of salt and pepper noise, but the method is only based on the feature and membership function, and does not consider the spatial feature information, which will lead to serious misclassification in complex water backgrounds. Therefore, it is necessary to develop an object-based and unsupervised extraction method that combines spectral features and spatial features.

Although the method based on line features can accurately delineate the locations of raft aquaculture in high resolution images (around 2 m), the lower spatial resolution of 15–30 m severely limits the use of such imagery mainly because linear features in a raft aquaculture area are not obvious at this resolution. Therefore, a fast and efficient extraction method that can employ low resolution remote sensing imagery is urgently needed for monitoring coastal raft aquaculture areas, which is of great significance for the rational development of marine resources and protection of the marine environment. At present, the extraction method of raft aquaculture areas using Landsat imagery has mainly been based on the spectral ratio method [26,27]. However, when some of raft aquaculture areas lie close to the deep sea in the spectrum or when the spectrum is not always uniform, these conditions will result in an inaccurate extraction result [28]. In the case of a complex seawater background, Wang [10] proposed a method for extracting raft aquaculture areas based on object-based visually salient NDVI features (OBVS-NDVI). The main principle of this method is based on the visual saliency calculation methods proposed by Itti [29] and Sun [30], which allows the highlighting of raft aquaculture areas by NDVI feature enhancement of segmentation objects. However, these methods still have a relatively low level of accuracy when only considering spectral information in some spectrally similar non-aquaculture zones, such as coastal shoals and so on. Introducing the spatial structure features, such as the edge feature, it not only retains the advantages of the OBVS-NDVI method to enhance the spectrum of marine aquaculture areas, but also improves the extraction effect in complex seawater backgrounds. And it would provide critical supplementary information to allow a better differentiation of natural water bodies and raft aquaculture areas.

With the goal of promoting the accurate extraction of raft aquaculture areas from medium resolution imagery (15–30 m) in a complex seawater background, we propose a new method based on our former method [10]. The newly proposed method not only makes use of the spectral characteristics of NDVI, but also integrates other spatial features, i.e. edge features. By comparing experiments with our previous OBVS-NDVI method at three sites aligned from north to south in the coastal areas of China, the improved extraction accuracy was confirmed.

## 2. Methods

**Figure 1:**
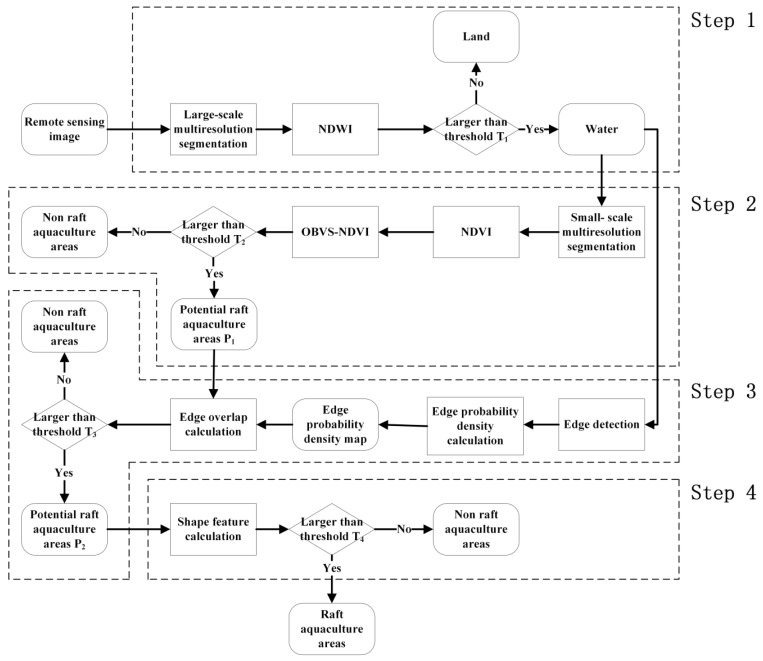
Workflow of the proposed raft aquaculture extraction method. Note: NDVI, Normalized Difference Vegetation Index; OBVS-NDVI, Object-Based Visually Salient NDVI; NDWI, Normalized Difference WaterIndex; P_1,_ the potential raft aquaculture areas extracted by OBVS-NDVI; P2, the potential raft aquaculture areas extracted by overlap degree of the edge and OBVS-NDVI features of the raft aquaculture area; T_1_, T_2_, and T_3_, represent three key initial thresholds in target segmentation: NDWI threshold T_1_ for water-land separation, OBVS-NDVI feature extraction for potential raft aquaculture area threshold T_2_, and edge overlap threshold T_3_; T_4_, the threshold value 1.5 times of the standard width of the patch in the actual raft aquaculture.

The new proposed method requires four main steps as shown in a technical flowchart (Figure 1). Step 1: Separate water and land by threshold segmentation of the normalized difference water index (NDWI). Step 2: Calculate the OBVS-NDVI and then extract potential raft aquaculture areas P_1_ based on the threshold. Step 3: Conduct edge detection first; then extract the potential raft aquaculture areas P_2_ by the degree of edge overlap and OBVS-NDVI features of the raft aquaculture area. Step 4: Reprocessing selection by shape feature. Details of these steps are described below.

### 2.1. Water Area Extraction

Although the NDVIs of raft aquaculture areas are higher than most water areas, they are still far lower than forestland or grassland and are even lower than bare land [10]. Therefore, it is necessary to separate water from land; only for water areas is it helpful to enhance the NDVI target to gain an accurate extraction of raft aquaculture areas. McFeeters [31] has proposed that using NDWI can effectively enhance water targets. In this paper, we use NDWI to separate water and land using Equation (1):

(1) NDWI=(G−NIR)/(G+NIR)

where for Landsat 8 Operational Land Imagery (OLI), *G* is the mean value of the green band and *NIR* is the mean near infrared band.

When the NDWI index is greater than the threshold value of $T_{1}$, the segmented patches are delineated as water body area, and the other patches are land. The threshold for setting of the $T_{1}$ criterion allows researchers to distinguish the water area from the land in an image. The Otsu’s method can be used to determine the threshold [32]. Otsu’s algorithm assumes that the image pixels can be divided into two parts, the background and the target according to the threshold. Then, the optimal threshold is calculated to distinguish the two types of pixels, so that the two types of pixels have the largest degree of discrimination. It is an adaptive threshold determination method.

### 2.2. Potential Raft Aquaculture P_1_ Extraction by Thresholding OBVS-NDVI

Although the *NDVI* [33] in coastal raft aquaculture areas have very distinctive characteristics, some seawater areas maybe have high *NDVI* values because of their complex spectral characteristics, which will result in inaccurate and excessive extraction of coastal raft aquaculture areas. Therefore, in the extraction of raft aquaculture area, we can calculate the salient *NDVI* characteristics [10] of each patch in the water area based on Equations (2) and (3), and delineate the raft aquaculture surface target areas:

(2) NDVI=(NIR−R)/(NIR+R)

(3) $$S_{O}\left(NDVI\right)=\frac{\sum_{O_{j\in N\left(O\right),m_{O_{j}}\left(NDVI\right)<m_{o}\left(NDVI\right)}}B\left(O,O_{j}\right)\left(m_{O}\left(NDVI\right)-m_{O_{j}}\left(NDVI\right)\right)}{\sum_{O_{j}\in N\left(O\right)}B\left(O,O_{j}\right)}$$

where for Landsat 8 OLI imagery *NIR* represents the mean near infrared band and *R* represents the mean red band; *N(O)* represents the set of the neighboring objects of *O*; $B\left(O,O_{j}\right)$ represents the common boundary length between object *O* and object $O_{j}$; and $m_{O}\left(NDVI\right)$ represents the mean *NDVI* of object *O*. Note that a constraint of $m_{O_{j}}\left(NDVI\right)<m_{O}\left(NDVI\right)$ exists under the summation symbol in the numerator; $S_{O}\left(NDVI\right)$ represents the visually salient feature of the current object *O*, i.e., OBVS-NDVI. In this step, we set the potential raft aquaculture areas P_1_ in the water with the characteristic OBVS-NDVI greater than threshold *T_2_*, while the other areas are set non-aquaculture areas.

### 2.3. Potential Raft Aquaculture P_2_ Extraction by Thresholding Edge Overlap

**Figure 2:**
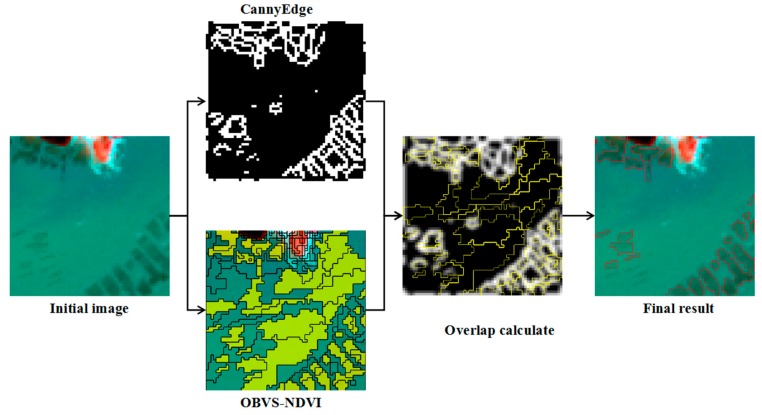
Schematic diagram of overlap calculation.

The diagram of raft aquaculture extraction based on edge overlapping is shown in Figure 2. First, Canny edge (https://en.wikipedia.org/wiki/Canny_edge_detector) and OBVS-NDVI features are extracted from the initial image, and then they are overlapped. The overlap probability of edge pixels between Canny edge and OBVS-NDVI features is calculated, and the regions with a degree of overlap greater than *T*_3_ are identified as potential raft aquaculture areas P_2_. Details of these steps are described below.

In this paper, we first use the Canny edge extraction algorithm to extract the edge of the coastal raft aquaculture area in the water areas [34,35]. The edge is recorded as a two-dimensional grid matrix, where an edge value is 1 and a background value is 0. Then the OBVS-NDVI characteristics of the water area segmentation are processed. Suppose the patch of OBVS-NDVI feature is $S_{O_{j}}$, where j∈J, and *J* is the number of patches with OBVS-NDVI features. At this time, the OBVS-NDVI surface primitive patches is searched to find the edge and marked as $B_{j}$, indicating four neighborhoods where the target belongs to the boundary of a polygon element feature patch but does not belong to its boundary pixel, which is recorded as:

(4) $$B_{j}=\{p|p\in B_{j}\forall N_{4}\left(p\right)\notin S_{O_{j}}\}$$

where, for a pixel p(x,y) in the image, the coordinates of the four pixels adjacent to it in the horizontal and vertical directions are respectively (x−1,y), (x+1,y), (x,y−1), (x,y+1), Then these four pixels constitute the four domains of the pixel p(x,y), which are expressed as $N_{4}\left(p\right)$.

Nevertheless, for the Canny edge feature of edge detection in the water area, we want to produce an edge probability map; therefore, we use the Gauss filtering method. Gauss filtering is a process of weighted average of the entire image [36], where a template shown in Equation (5) is used to scan every pixel in the image, and the weighted average gray value area of pixels in the domain determined by the template is used to replace the value of the center pixel point of the template. The template size selected in this paper is 3×3:

(5) $$g\left(x,y\right)=\frac{1}{2\pi \sigma^{2}}e^{-\frac{x^{2}+y^{2}}{2\sigma^{2}}}$$

where (*x*, *y*) represent the point coordinates for pixels, and σ represent the standard deviation of the Gauss filter. Through the Gauss filtering of the edge map, we obtain the edge probability map $E_{g}\left(x,y\right)$.

The accurate extraction of raft aquaculture areas is realized by combining the edge feature with the object patch feature. It is realized by summing up the edge probability graph, and then calculating the mean probability value between them. The calculation of edge overlap is shown in Equation (6):

(6) $$R_{j}=\frac{1}{\left|B_{j}\right|}\underset{p\in B_{j}}{\sum }E_{g}\left(x,y\right),$$

where $E_{g}\left(x,y\right)$ is the edge probability graph; $\left|B_{j}\right|$ is the sum number of pixels of $B_{j}$; and $R_{j}$ is the average of the overlap probability between the edge feature and OBVS-NDVI feature. The threshold of the overlap is denoted as *T*_3_. Then, we set the areas in the water with the overlap degree greater than *T*_3_ as the potential raft aquaculture areas *P*_2_, while the other areas are non-aquaculture areas.

### 2.4. Reprocessing Extraction by Shape Feature

The last step is to do feature post-processing of the aspect ratio shapes. In comparing the statistical results with the actual situation, we set 1.5 times the standard width of a patch in actual raft aquaculture as the threshold value *T*_4_. Next, we set the areas in the potential raft aquaculture areas P_2_ with a patch width greater than *T*_4_ as the non-aquaculture areas; the others are final raft aquaculture areas. The relative width of the threshold (*T*_4_) can be roughly estimated through the calculation results of Equation (7). The patch set in this value range represents the target raft aquaculture areas. The relative width of the patch waiting to be processed can be calculated using Equation (7):

(7) $$W=\frac{2A}{\left|B_{j}\right|}$$

where *A* is the area of the current patch $O_{j}$ represented by the number of pixels being processed, and $\left|B_{j}\right|$ represents the number of elements in the edge the pixels of $O_{j}$.

## 3. Experiments and Analysis

### 3.1. Experimental Data

**Figure 3:**
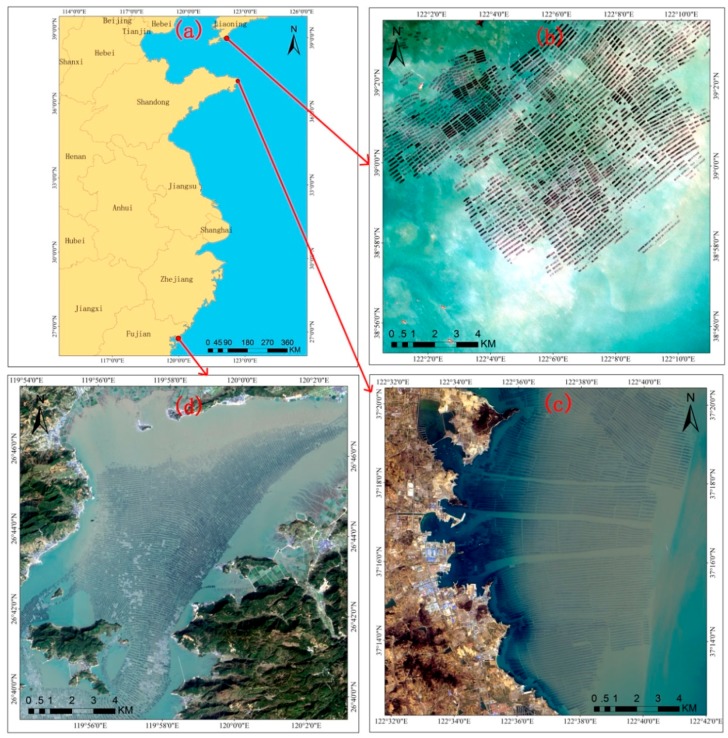
Map of (**a**) coverage of the experimental area and aerial photographs of study areas (**b**) 1, (**c**) 2, and (**d**) 3.

As described in the introduction, raft aquaculture in the Asian region accounts for 90% of the global total. Among them, coastal raft aquaculture in Liaoning, Shandong and Fujian has a wide range and high density, and the environmental impact is more prominent. Moreover, China has a wide latitude range. The selected three experimental areas have certain differences in factors such as climate and hydrological conditions, and are suitable for testing the stability and reliability of the extraction algorithm in aquaculture areas. Therefore, this paper selects these three experimental areas from north to south in China (Figure 3). The experimental data were acquired from USGS (http://glovis.usgs.gov/). Study areas 1, 2, and 3 are located near Liaoning, near Shandong, and near Fujian, respectively (Table 1).

In this paper, we randomly selected three Landsat 8 OLI image datasets to describe the events in the real world of coastal raft aquaculture area. The original Landsat remote sensing image processed by this method had a multi-spectral resolution of 30 m and panchromatic band resolution up to 15 m. Nine bands were selected including Coastal (0.43–0.45 μm), Blue (0.45–0.51 μm), Green (0.53–0.59 μm), Red (0.64–0.67 μm), Near infrared (0.85–0.88 μm), Panchromatic (0.50–0.68 μm), and Cirrus (1.36–1.38 μm) bands as well as Short infrared bands 1 (1.57–1.65 μm) and 2 (2.11–2.29 μm). Before the experiment, image preprocessing included image cutting, fusion, and so on, so that the spatial resolution of the experimental images was 15 m after processing. The performance of different methods for extracting coastal raft aquaculture area was studied, including the proposed method introduced in this paper.

### 3.2. Accuracy Evaluation

Commonly used performance measures [12], including *Recall*, *Precision*, and *F-Measure*, were employed to evaluate the accuracy of the method employed here using Equations (8)–(10), respectively:

(8) $$Precision=\frac{TP}{TP+FP}$$

(9) $$Recall=\frac{TP}{TP+FN}$$

(10) $$F-Measure=\frac{2\times Recall\times Precision}{Recall+Precision}$$

where *TP* is the number of pixels that were correctly extracted, *FP* is the number of pixels that were not extracted, *FN* is the number of pixels that were misidentified, and *F-measure* is a combination of the *Precision* and *Recall* performance measures calculated as a harmonic mean of the two above measures. An ideal raft aquaculture extraction method should have high *Precision* and *Recall* ratios. In practice, however, *Precision* and *Recall* measures will conflict with each other. Thus, *F-Measure* was used as the comprehensive index to evaluate the performance of the method used in our experiments.

### 3.3. Parameter Setting

#### 3.3.1. Image Segmentation Parameters Setting

This experiment used the Multiresolution Segmentation (MRS) algorithm segmentation method [37,38]. This algorithm includes three main parameters: *shape*, *compact*, and *scale*. The range of values for the *shape* parameter is [0,1], which was mainly used for the balance of spectral and shape features during image segmentation. The smaller the *shape* value, the more consistent the segmentation objects are in spectral features. Similarly, the range of values of the *compact* parameter is [0,1], which was mainly used to control the shape of the segmentation object. The larger the *compact* value, the closer the segmentation object will be to a square in shape. *Scale* is a parameter that controls the size of the split object. The equation with details can be seen in [37,38]. Because spectral features still dominate the extraction in a raft aquaculture area, we set the *shape* parameter at 0.1, which is recommended by default for MRS. In addition, the *compact* parameter was set at 0.5 by default, because raft aquaculture areas were banded in most cases and varied in length.

The *scale* parameter that controls the size of segmentation has a great influence on the extraction results [39,40]. Setting *scale* too small will resulted in the segmentation objects becoming too fragmented, making it easy to produce a similar pixel based classification of “salt and pepper noise” in the results. However, if *scale* is set too large, this will easily cause the target and background to become part of the same patch, making it difficult to distinguish the targets in subsequent extraction steps. Many research studies have been carried out by predecessors attempting to choose an appropriate scale; these mainly resulted in two methods: the supervision method using reference segmentation imagery [41] and the non-supervision method that does not use reference segmentation imagery [42]. Here, we referred to Wang et al. [43] who proposed the logarithm of the segmented object and the logarithm of the scale parameter have a linear relationship that can be used to calculate the optimal segmentation scale. Finally, the patch segmentation *scale* of raft aquaculture areas was set as 20 in the present study, while the segmentation *scale* of land and water separation was set as 300.

#### 3.3.2. Threshold Parameters (*T*_1_, *T*_2_, *T*_3_) Setting

Based on the description of the second part, our method involves the following three important initial thresholds used during target segmentation: NDWI threshold *T*_1_ for water-land separation, OBVS-NDVI feature extraction for potential raft aquaculture area threshold *T*_2_, and edge overlap threshold *T*_3_. Considering that water extraction is the scope of the limited raft aquaculture area, and there are further processing steps, the water extraction threshold *T*_1_ based on NDWI is uniformly set to 0 here to extract as much water as possible. This is because there are additional mechanisms (include OBVS-NDVI feature extraction, edge overlap screening, shape feature extraction mechanisms) used in raft aquaculture areas, and a small number of errors in the initial extraction results have little effect on the subsequent extraction results. Wang et al. [10] used OBVS-NDVI features to obtain the best threshold by applying the *F-Measure* maximum. However, the threshold might change when the data sources, regions, and data acquisition times are different, making it difficult to popularize the threshold method in practice because drawing reference data requires a great amount of time. Therefore, we can set a small threshold when using OBVS-NDVI features to extract potential targets in our method, because there will be an edge overlap screening mechanism later. Here, we set the threshold *T*_2_ to 0 because the raft aquaculture area must be relatively distinct from the surrounding area. The present study focused on the degree of edge overlap; we will make a comprehensive analysis of *Precision*, *Recall*, and *F-Measure* in Section 3.4.1.

### 3.4. Result and Analysis

#### 3.4.1. Edge Overlap Degree Experiment of Different Threshold

**Figure 4:**
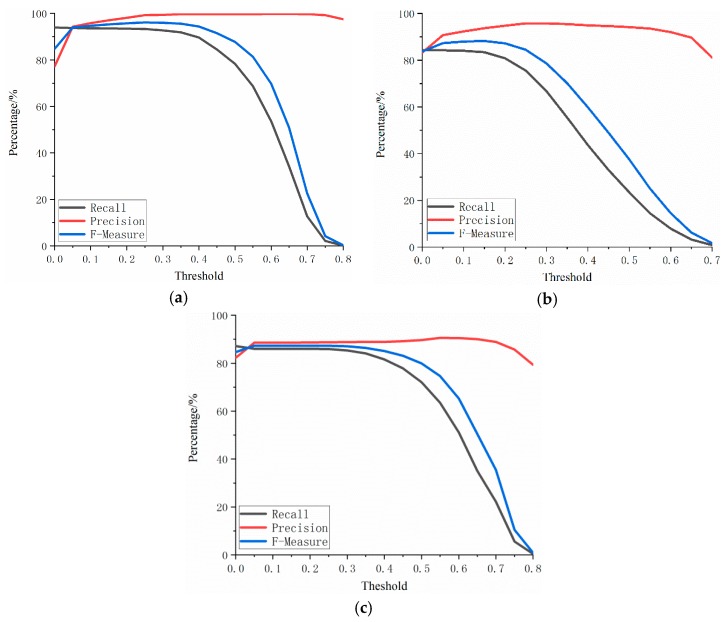
*Precision* analysis of threshold selection of the degree of overlap in raft aquaculture areas: degree of edge overlap in experimental result of study areas (**a**) 1, (**b**) 2, and (**c**) 3.

The threshold of the optimal degree of overlap was first determined through the experiment. We selected a threshold value between 0 and 1 in each interval of 0.05 for the experiment (Figure 4). The following phenomena can be seen from the experimental results in the three areas.

(1) *Precision* remained relatively stable and basically stayed above 80%. The response of *Precision* resulted in the correct extraction ratio in the extraction result. Therefore, the “overall stability” indicates that the introduced edge overlap elimination step retains the correctly extracted raft aquaculture area. In addition, the basic maintenance above 80% indicates that the OBVS-NDVI method can accurately identify raft aquaculture areas.

(2) The overall trend of *Recall* is designed to first maintaining stability and then gradually decreasing. The *Recall* value reflects the proportion of the extracted results in all of the actual raft aquaculture areas. When the exclusion method has no effect, that is, when it is in a random state, the *Recall* value is roughly constant before it reduces beyond a threshold value. As can be seen here, *Recall* has a period of stabilization before reduction, which indicates that when the threshold value of the degree of edge overlap introduced is raised, some non-aquaculture areas are basically excluded, while the correctly extracted raft aquaculture areas are retained. In addition, the proportion of correctly extracted areas among all actual aquaculture areas remains unchanged.

(3) For the three raft aquaculture areas analyzed in the present study, the proportion of *Precision* was basically higher than *Recall*. Combined with the meaning of these two indicators, we can see that this trend reflects the fact that the method used with the above parameters creates slightly more serious leakage of the raft aquaculture areas. Of course, this situation can be improved by selecting appropriate thresholds for OBVS-NDVI feature extraction, but it should be noted that this operation is also likely to reduce the *Precision*.

(4) A remarkable feature is that a steep rise in *Precision* was observed at the position of about 0.05–0.1, and in *F-Measure* indicators of the three regions; later, these maintained a stable period, after which the *F-Measure* index gradually declined. This stage of a steep rise with the edge overlap threshold reflects the method of edge overlap, which can exclude a large number of non-aquaculture areas while retaining the correctly extracted raft aquaculture areas. The stationary period indicates that, whether it is erroneously or correctly extracted during the process of increasing the threshold, an area is not excluded. It indicates that this stationary period is the limit of the highest accuracy that can be achieved by the method in this parameter adjustment range. It is also the selection range when the edge overlap threshold is set. If the threshold of degree of edge overlap is lower than this range, then the non-aquaculture areas cannot be fully excluded. However, some raft aquaculture area will be incorrectly excluded if this range is exceeded resulting in serious omissions.

The analysis of the above four phenomena illustrates a fact that the edge of overlapping effect is significantly improved when using the OBVS-NDVI method.

#### 3.4.2. Comparative Method Analysis

**Figure 5:**
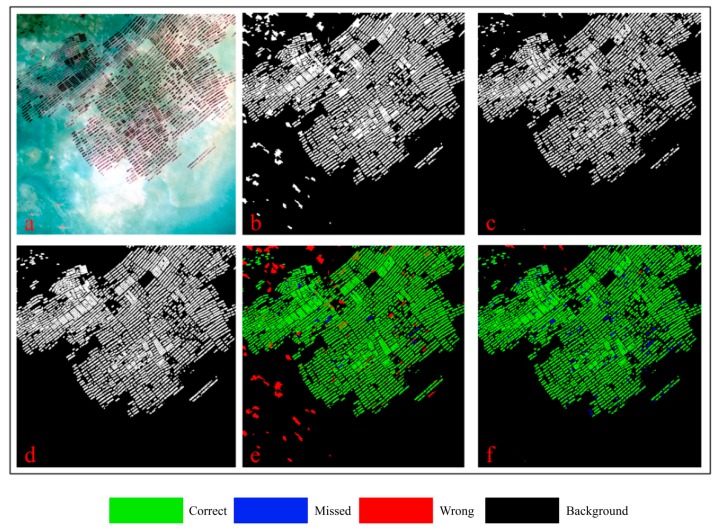
Results of two experimental methods for study area 1: (**a**) Initial image; (**b**) OBVS-NDVI extraction result; (**c**) Proposed extraction result; (**d**) Human interpretation result; (**e**) Overlap of OBVS-NDVI result with human interpretation result; (**f**) Overlap of proposed result with human interpretation result.

**Figure 6:**
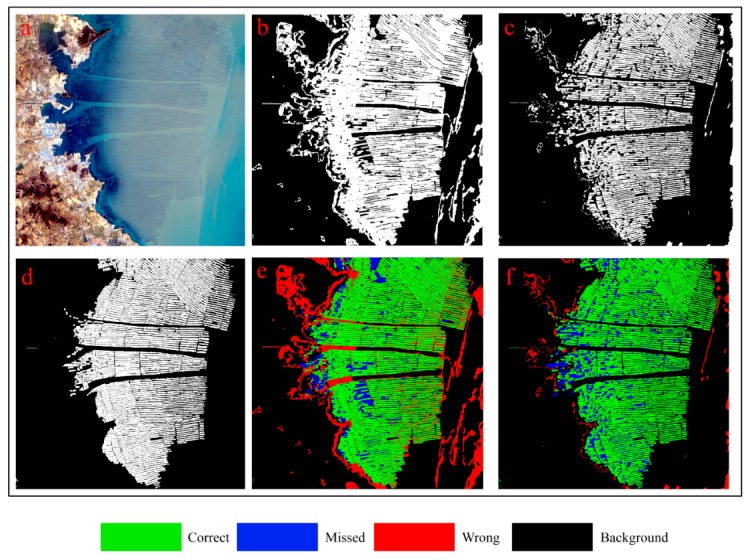
Results of two experimental methods for study area 2: (**a**) Initial image; (**b**) OBVS-NDVI extraction result; (**c**) Proposed extraction result; (**d**) Human interpretation result; (**e**) Overlap of OBVS-NDVI result with human interpretation result; (**f**) Overlap of proposed result with human interpretation result.

**Figure 7:**
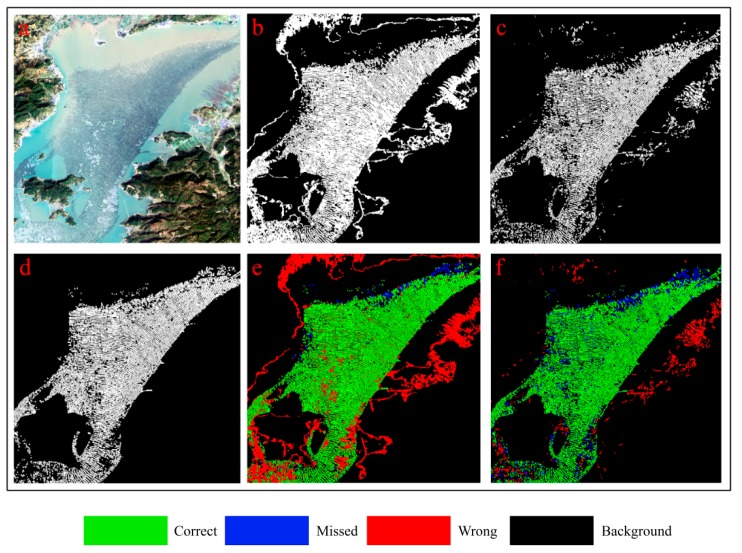
Results of two experimental methods for study area 3: (**a**) Initial image; (**b**) OBVS-NDVI extraction result; (**c**) Proposed extraction result; (**d**) Human interpretation result; (**e**) Overlap of OBVS-NDVI result with human interpretation result; (**f**) Overlap of proposed result with human interpretation result.

The results of the previous Section 3.4.1 provide only quantitative comparisons in the statistical sense. Determining the superiority of the OBVS-NDVI method or our method in spatial distribution proved difficult. Therefore, we used a maximization of the *F-Measure* to compare the extraction results. With the overlap thresholds of study areas 1, 2, and 3 set at *T*_3_ = 0.25, 0.15, and 0.25, the results are shown in Figure 5, Figure 6 and Figure 7, respectively; the related data can be seen in Table 2. To avoid possible sampling errors, validation data covering the entire image was generated via human interpretation. In addition, we also checked the google earth high resolution image of the uncertain area about the same period or similar period to ensure the accuracy of the human interpretation result.

Based on comparing the results shown in Figure 5, Figure 6 and Figure 7 and Table 2, we can see that the accuracy of the proposed method is greatly improved by introducing the spatial feature of edge on two following aspects:

(1) The comprehensive accuracy is significantly improved compared with the OBVS-NDVI method. According to the F-Measure (described in Section 3.2) values of the three experimental areas in Table 2, the method proposed in this paper has a higher accuracy in extracting coastal raft aquaculture areas. It also can be seen from Figure 5, Figure 6 and Figure 7 that the area extracted correctly by the method in this paper is significantly increased, as shown in the green part of the figure. The accuracy of this method is about 96% when the seawater background is relatively uniform, such as area 1 (Figure 5). In addition, the accuracy of this method is about 10% higher than that of OBVS-NDVI method when the seawater backgrounds become complex in areas 2 and 3. The robustness of the proposed method is proven by the extraction of aquaculture areas under the background of the three experimental regions analyzed here. 

(2) The correct extraction is greatly improved although the omissions increase lightly compared with OBVS-NDVI method. The OBVS-NDVI method only uses the spectral information of the image to have errors. This paper improves the extraction effect in the complex background of the coastal raft aquaculture area based on the OBVS-NDVI by introducing the spatial feature—edge information. By comparing the *Precision* and *Recall* indices, we can see that although the *Recall* index of the method is slightly lower in this paper, the *Precision* index is greatly improved. From the extraction results of area 2 (Figure 6) and area 3 (Figure 7), we can see that the fault-prone phenomenon in coastal raft aquaculture areas with large differences in seawater spectral variation based on OBVS-NDVI method is serious, especially when the islands of seawater are complex. In addition, a large number of tidal flats next to some islands were mistaken as raft aquaculture areas, which are noted in red in the figures. However, these errors were eliminated by the method of edge overlap degree, and further removed by shape feature post-processing. This makes the misclassification phenomenon significantly reduced compared with OBVS-NDVI method.

There are some misclassifications and omissions in the proposed method. The misclassification part is mainly because the spectrum and morphology of some dotted high chlorophyll concentration area are similar to those of raft aquaculture areas. And the omission part is mainly due to the fact that the raft aquaculture area is in the growing season, and its spectral characteristics and edge characteristics are not obvious. And in the post-processing, it is easy to treat some large plaques that are connected together in the segmentation process as non-target culling, which also causes the omission phenomenon.

## 4. Summary

We have presented a new method for raft aquaculture extraction from medium resolution (15–30 m) satellite images and experimented using Landsat 8 OLI images. The proposed method integrates both spectral and spatial features, i.e., NDVI and edges. A comparison of experiments with our former object-based visually salient NDVI method on three sites confirmed that the accuracy has been obviously improved in water areas with complex backgrounds. Considering the freely accessible data source, the method holds a great potential for rapid monitoring of coastal raft aquaculture on a large regional scale.

Whatever, this method still has some the shortcomings: (1) There are many threshold parameters to be set. When using this method, it is necessary to clearly understand the meaning of each parameter. The best way is to perform manual checks and adjustments during each step of the process; for example, threshold settings for canny edge detection, scale segmentation, water and land separation, and so on. (2) The method only focused on the coastal raft aquaculture area. And the extraction of other types of aquaculture areas such as pond and cage aquaculture remains to be further studied. (3) The test experiment of this method only selects the areas in China’s coastal areas from Landsat 8 OLI. In future studies, we will consider additional types of aquaculture areas, and carry out regional application research on both regional and temporal dimensions.

## Figures and Tables

**Table 1:** Introduction to the experimental areas.

Name	Location	Size	Acquisition Time	Image Path/Row
Area 1	Liaoning	1023 × 1023	03MAR2017	119,033
Area 2	Shandong	1026 × 1026	21MAR2016	119,034
Area 3	Fujian	1025 × 1025	13FEB2017	118,041

**Table 2:** Accuracy evaluation results of the three study areas.

Study Area	Method	*Recall* (%)	*Precision* (%)	*F-Measure* (%)
Area1	OBVS-NDVI	96.22	87.35	91.57
	Proposed	93.30	99.26	96.19
Area2	OBVS-NDVI	89.65	66.85	76.59
	Proposed	83.40	93.68	88.24
Area3	OBVS-NDVI	95.67	60.90	74.43
	Proposed	85.90	88.74	87.30

Note: OBVS-NDVI, object-based visually salient normalize difference vegetation index.
